# Understanding the mind-brain relationship through focal neurodegenerative pathology

**DOI:** 10.1007/s10072-025-08380-8

**Published:** 2025-08-20

**Authors:** Maria Caterina Silveri

**Affiliations:** Department of Psychology, Catholic University, Largo A. Gemelli, 1, Milan, 20123 Italy

**Keywords:** Mind brain relationship, Cognitive models, Neuroimaging, Vascular damage, Neurodegeneration, Semantic dementia, Primary progressive apraxia of speech

## Abstract

This paper considers the contribution that observations of neurodegenerative diseases can make to our understanding of the ‘mind-brain’ relationship. The theoretical context in which cognitive models have been implemented by evidence from brain-damaged patients and the contribution of neuroimaging are briefly described. Reasons why neurodegenerative pathologies, as ‘systems’ pathologies, are potentially useful in providing complementary information to that obtained in focal vascular pathologies are reported and two specific examples, semantic dementia and primary progressive apraxia of speech, are discussed. While recognising the role of functional neuroimaging, priority should be given to the study of patients with brain damage, especially of a neurodegenerative nature, to legitimise the relationship between specific functions and specific structures (systems).

## Cognitive neuropsychology and neuropsychological models

The mind-brain relationship is the point of convergence between the two groups of disciplines within cognitive neuroscience, those derived mainly from the medical and biophysical sciences, and those derived from computer science and philosophy (such as the psychology of information processing, linguistic and artificial intelligence) [1, 2]. In recent decades, there has been an extensive debate about the methodologies that should be used to study this relationship. Within the framework of the so-called ‘computational’ models [3], to which classical cognitive neuropsychology refers, based on ‘representations’ and ‘processes’, cognitive neuropsychology has been accused of focusing mainly on processes, with descriptions of the flow of information transformation, and neglecting the level of representation, with the real aim of diagramming the functional architecture of cognitive processes, without worrying too much about explaining how the various subcomponents (boxes and arrows) of the diagram work [4]. The clear preponderance of observations made on single brain-damaged subjects was justified by the intention to overcome the limitations of inter-case variability of group studies (brain lesions in different patients can never be identical) [5], where the lack of homogeneity of the group could make the mean performance indicative of the behaviour of only some, or even none, of the subjects [6].

The single case approach is based on the assumption, not always explicitly stated, that cognitive abilities have the same functional organisation in all brains, at least in the same cultural context, and also that all brains are organised in the same way, so that observations from single cases can be extended to the general population [7]. There would be a correspondence between cognitive modules and specific brain regions, and the diagram representing the functional organisation of different cognitive abilities would have a correspondence in neural structures, with the boxes representing the modules and the arrows the neural connections between modules [8] (see [9] for discussion).

The representation of cognitive models in diagrams is based on the assumption that the human mind, like all complex systems, has a *modular* organisation [10–14]. Since Gall’s early observations that individual mental functions are located in discrete regions of the brain (reviewed by Simpson [15]), the concept of modularity has influenced studies of the mind-brain relationship; however, the greatest influence seems to have been on cognitive neuropsychology. Although there is no complete agreement on the definition of modules, they are currently conceived as a set of separate domain-specific components supported by specific brain areas; modules are computationally independent of other modules, genetically determined and encapsulated - i.e. independent of top-down influences - with a specific developmental trajectory. The organisation of modules is essentially ‘vertical’ in the sense that modules do not interact with each other except to pass processed information to the next module.

*Double dissociation* [13], i.e. the demonstration that a subject (or group of subjects) with a given cerebral lesion, performs significantly better on Task A than on Task B, whereas another subject (or group of subjects) with a lesion at a different site, shows the opposite pattern (B performs significantly better than A), has been one of the cornerstones of cognitive neuropsychological methodology. The basic assumption is that impaired performance on tasks A and B reflects a deficit in the subcomponents of the cognitive system that the tasks probe, and that the abilities being probed are somehow correlated with the location of the respective lesions. Early observations that pioneered the so-called method of anatomoclinical correlation, typically based on the description of behavioural changes in neurological patients with lesions in specific brain regions, were implicitly based on the principle of double dissociation. The prototype of the methodology of anatomoclinical correlation between symptoms and lesion localisation is in the observations reported by Broca and Wernicke in the second half of the 1800s, i.e., posterior inferior frontal and posterior temporal regions of the left hemisphere are associated with language production and comprehension disorders, respectively. Thus, if lesions of the posterior inferior frontal regions impair language production but not language comprehension and lesions of the posterior temporal regions impair comprehension but not production, it can be inferred that comprehension and production are independent functions and independent are the respective neural substrates. Thus, although the single-case approach of cognitive neuropsychology has been considered by some to be in decline [16], it should at least be acknowledged that it gave rise to modern neuropsychology and to studies of the relationship between mind and brain based on the scientific method.

While moving away from the extreme positions held by some neuropsychologists, according to which knowledge of the physical (neural) basis is largely irrelevant to the implementation of cognitive models that can only be based on behavioural analysis of cognitive dysfunction [17], it is a fact that the methodology of research with brain damaged patients has been largely influenced by the assumptions of cognitive neuropsychology [18]. This has certainly favoured the construction of increasingly sophisticated models of the functional organisation of cognition, which have not been matched by an equally detailed knowledge of their respective neural bases. In other words, neuropsychologists have long pursued the methodology of double dissociation, reducing the functional organisation of cognitive processes to an increasingly complex and detailed set of modules (boxes and arrows) without an equally strong interest in the neural correlates. The study of syndromes that cite associations between symptoms rather than dissociations, and that consider interactions rather than modularity, especially when confirmed by case series observations [19], has long been considered only ‘complementary’ to the implementation of models of cognitive functioning.

As a result, until the end of the last century, neuropsychological research contributed more to the construction of functional models of behaviour than to the understanding of the neural basis of behaviour. This was also justified by the fact that neuroimaging data, at that time mostly based on computed axial tomography, did not provide sufficiently detailed information. In conclusion, with a few exceptions, neural models, at least those derived from the observation of pathological behaviour in neurological patients, have not deviated much from those proposed by the classical observations of the 19th/early 20th century.

## Neuroscience for neuropsychology: the contribution of neuroimaging to the study of the mind-brain relationship

Although the correspondence between cognitive modules and specialised brain areas has often been taken for granted in the neuropsychological literature, the hypothesis of the functional organisation of the brain in terms of “modularity”, as well as the question of the correspondence between functional cognitive components and brain structures, has never been explicitly advanced until the introduction of functional neuroimaging. The concept of ‘segregation’ (or ‘modularity’) of specialised brain areas, as well as that of functional ‘integration’ between different brain areas as the basis of brain organisation, derives principally from functional MRI studies [20], (see for review [21]). At the same time, the development of structural neuroimaging techniques, in particular structural MRI, has made and continues to make an important contribution to the study of the mind-brain relationship in lesion studies, by providing an ever sharper definition of lesion location and extent and of white matter bundles connecting different cerebral regions.

The anatomo-clinical method of linking brain damage to cognitive impairment has gradually been complemented (and replaced) by functional neuroimaging observations in normal subjects. Just as in the lesion study the cognitive deficit is the result of a reduction in resources in a particular brain region, the activation of brain regions in normal subjects is the result of the resources required by those particular regions to process the information relative to the task that the subjects are performing.

Despite the high expectations generated, functional studies (mainly task-related fMRI) have also attracted several criticisms on both technical and theoretical grounds. For example, the Brain Oxygen Level Dependent (BOLD) signal on which task-related fMRI is based is an indirect measure of neuronal activity [22]; moreover, it is not always transparent which cognitive components underlie the task and thus to which cognitive process the activation should be related. Studying the neural mechanism underlying the task, without knowing the structure and computational models of the processes underlying the experimental task used, is like “putting the cart before the horse” ([23] p. 42). Without sharing the pessimistic conclusion that activation studies do not tell us much about the neural mechanisms underlying cognitive functions, at the risk of returning to a new phrenology [24], the purpose of functional neuroimaging studies should go far beyond the (re)construction of sophisticated and detailed functional maps. As discussed by Price [25], the relationship between cognitive abilities and brain localisation that emerges from functional neuroimaging studies is not a one-to-one relationship, that is, the same region can be associated with different functions and the same function can be associated with different brain regions. This suggests the need for a many-to-many mapping between cognitive functions and brain structures, on the basis of which new functions from structures and structures from functions could be predicted, i.e. new functional ontologies for cognition [26]. Without diminishing the contribution of neuroimaging data to cognitive models, to which metanalytic studies of ‘big data’ could also contribute [27], well-specified behavioural models based on evidence from brain-injured patients should given epistemological priority over neuroimaging to explore the mind-brain relationship without reductionist conclusions [28–30].

## Lesion methodology: vascular lesion vs. neurodegeneration

The brief introduction has attempted to highlight the advantages, but also the critical aspects, of lesion methodology in the context of cognitive neuropsychological models and observations derived from functional neuroimaging in the study of the mind-brain relationship. Neurodegenerative pathologies may provide some suggestions in this regard. As a first observation, it should be noted that certain brain structures, such as the left temporal pole, tends to be spared from ischemic damage for hemodynamic reasons, with the result that it has only recently been included in the language network, thank to observations in neurodegenerative pathologies such as semantic dementia (SD) [31]. Similar considerations apply to prefrontal regions and the right anterior temporal lobe, whose dysfunction in frontotemporal dementias is a privileged target of study for understanding the neural basis of behaviour.

It has already been mentioned that a critical aspect in the study of the mind-brain relationship is the fact that brain lesions of a vascular nature cannot be considered identical in different patients [5], which limits the reliability of the results obtained from groups [6]. The non-comparability of vascular brain injuries is an objective fact. It is virtually impossible, for the extent of lesions in the cortex and subcortical structures and the involvement of white matter bundles and possible disconnection with distant cortical and subcortical structures, for the damage of different subjects to overlap. Furthermore, the boundary of vascular damage, which is related to the structural relationship between the vascular system and the brain structures and the level at which the vascular occlusion occurs, is unlikely to respect the boundary of a functionally ‘segregated’ or ‘modular’ [20] area specific to a given function.

The ‘non-specificity’ of brain regions, according to which cognitive impairment after brain damage is not limited to a single cognitive deficit [25], may thus also be related to the large-scale involvement of functional and structural connectivity associated with the virtually systematic presence of white matter damage [32], which produces lesion patterns that are not always predictable and identifiable.

Instead, neurodegenerative pathologies are typically ‘systems pathologies’, characterised by the hierarchical involvement of specific functional systems and anatomical regions for the prion-like spread of misfolded proteins [33], organised at the microstructural, macrostructural and functional levels [34], with damage to specific neuronal subpopulations [35]. Neurodegeneration is not random, rather, involves large-scale, distributed networks that are specific to different diseases. This results in circumscribed atrophy within functional networks that correspond to anatomically predictable networks in the healthy human brain [36].

The observation of subjects with the same neurodegenerative pathology, presumably involving the same networks and, where identifiable, the same neuronal populations, justifies the search for a correlation between behaviour and structural basis, overcoming some of the limitations of vascular pathologies. The patterns of behavioural deficits consistently observed in subjects suffering from the same neurodegenerative pathology are unlikely to be casual, i.e. due to the random involvement of adjacent territories/modules supporting the respective processes.

Neurodegenerative pathology also provides a strong legitimacy for the valorisation of the association between deficits, i.e. the *syndrome*, and makes it possible to overcome the fundamental criticism levelled at the “associations” of classical cognitive neuropsychology, which aimed to build cognitive models exclusively on the basis of (double) dissociations. The neurodegeneration of specific systems helps to better define or redefine the concept of the module at the neurofunctional level, in so far as given deficits have a necessary correspondence in degraded networks that constitute the structural basis of the corresponding functions. The evolution of neurodegenerative pathologies, which are typically characterised by the progressive and essentially parallel involvement of functionally related systems and subsystems, also makes plausible the horizontal interaction between modules, rather than vertical flow of information, and thus the emergence of associations between symptoms.

Better than focal vascular diseases, neurodegenerative diseases facilitate the collection of cases with common characteristics, *case-series*, not only in the variables under study, but also in the overall cognitive picture, with a selection criterion represented by the type of disease in this case. The assessment of associated symptoms can provide indications of the interaction between distributed modules/systems operating in parallel, especially if supported by functional, structural and connectivity neuroimaging studies [37– 38] and by knowledge of the neuropathological processes characterising specific diseases and vulnerability of subsystems [39]. At the same time, the detection of possible differences allows for the control of variability between cases, which may be related to individual factors in the functional organisation of the neural basis or even to the different strategies used by individuals when performing cognitive tasks.

The observations reported, even during the evolution of pathology with the presumed progressive involvement of different hierarchically organised subsystems, can be compared with *computational models* based on the principles of parallel, distributed and interactive processes (see seminal work of Rumelhart and McClelland [40]). These models, in which the nature of the processes and representations are made explicit (see [4]for discussion), as opposed to information processing diagrams, are able to incorporate evidence from anatomy, neuropsychology, and functional brain imaging to hypothesise the architectural structure of cognitive systems, to simulate cognitive task performance, and, by making appropriate ‘lesions’ in the network, to produce the impaired behaviour found in clinical observation [41–42]. However, it is important to remember that although these models are neurally inspired, they refer to an abstract version of brain networks and lack direct neurophysiological references. They do however better reflect the overall flexibility of brain networks.

## Evidenze from semantic dementia and primary progressive apraxia of speech

### Semantic dementia

Three patients with a similar pattern of semantic impairment, probably semantic dementia (SD) (but then called dementia of ‘unknown origin’) (for a critical review of the “entity semantic dementia” see [43]) were described by Elisabeth Warrington in 1975 [44]. The three patients showed a similar progressive impoverishment of knowledge of objects and words, which occurred along hierarchical lines, affecting first the more specific and then the more general features of meaning. Another remarkable observation was that episodic memory and other aspects of cognition, particularly language, were preserved. This first report had captured the essence of the cognitive pattern of this pathology, confirmed by later descriptions [45–46], which consisted in the selective and progressive deterioration of semantic memory. The typical distribution of atrophy in the anterior inferior structures of the temporal lobes, coupled with the equally typical pattern of cognitive decline, was the starting point for the construction of one of the most influential neurobiological models of the organisation of semantic memory [47]. The semantic network is structured in the cerebral cortex respecting the anatomical organisation of the motor, sensory and language systems. The associative areas of the sensorimotor regions (called “spokes”) are connected to each other and to the temporo-parieto-occipital areas to construct cross-modal representations that combine information from different sensory modalities. The proximity of the temporal pole to the limbic regions and the orbitofrontal cortex would at the same time allow the combination of cortical sensorimotor and linguistic components with affective components, which is necessary for the construction of amodal abstract representations in a “hub” in the anterior temporal lobe [47]. The temporal pole thus becomes the hub of semantic organisation (see [48] for the relationship between the left and right temporal poles). In summary, the semantic knowledge would be supported by a distributed system in the cerebral cortex and limbic structures, and a network of connections provided by white matter bundles afferent to a hub in the anterior structures of the temporal lobe.

Although there are alternative conceptualisations of the neural basis of semantic memory that privilege inferior parietal regions as the basis of cross-modal semantic representations [49], the ‘semantic hub and spokes’ model remains the most robust among those hypothesising convergence zones for multimodal information and the progressive transformation of information into amodal abstract representations, just because it is strongly supported by the behaviour of patients with SD, combined with neuroimaging evidence. A recent study [50] has proposed a neurobiological model of temporal pole structure for the interpretation of naming and word comprehension, integrating evidence from neuroanatomical studies in primates and neuroimaging in humans. This region would be the confluence of visual, auditory and limbic streams at the end of the “what” pathway. A multilevel synaptic neural matrix rotating around three axes distributed in these regions of the left hemisphere would transform object and word representations from unimodal percepts to multimodal concepts. Non-verbal object recognition and behavioural control would be supported by the temporopolar region of the right hemisphere.

One aspect of SD that has not always been given due consideration is that temporal atrophy is not symmetrical in the two hemispheres during the early to intermediate stages of the disease. While the two temporal lobes are considered a single functional structure in some respects [48], the clinical presentation varies depending on whether atrophy predominates in the left or right hemisphere. These findings suggest the existence of distinct syndromes [51, 52], or at least different patterns of clinical expression, depending on the extent of involvement in the left or right hemisphere [53]. When atrophy prevails in the left temporal lobe, the most frequently observed case, cognitive impairment involves verbal semantics, presenting as anomia and comprehension disorders. When atrophy prevails in the right temporal lobe, the core feature is prosopagnosia [51, 54], which is associated with altered social behaviour [55]. Observations of SD patients with right temporal atrophy provide further insight into the mind-brain relationship. For example, they reveal the neural basis for processing social stimuli, such as faces, and its relation to control functions for congruent behaviour through connectivity between the anterior temporal and orbitofrontal regions [56]. SD patients with right-sided atrophy also highlight the role of the right hemisphere, particularly the right temporal regions, in processing emotional stimuli. The inability to process emotional stimuli, particularly those conveyed by the face, could significantly impact the social behaviour disorder typically observed in these patients. The face is not only fundamental for identifying people, but also for decoding their emotional state (see [57], for discussion).

Neuroimaging has been essential to implement neurobiological models of semantic memory in providing information on the distribution and progression of atrophic processes in both grey and white matter bundles by comparing structural and functional data [58]. The possibility of identifying patterns of biological and genetic markers could also shed light on subpopulations of subjects whose symptoms may be related to the specific vulnerability of neuronal subpopulations and networks, again potentially correlated with behavioural data [39, 50] (see for example the specific vulnerability of the temporal pole network to the TDP-43 type C protein pathology) [50, 59].

Finally, the series of observations reported in subjects with SD confirms the existence of modules/functions related to specific neural systems. The consistent and systematic association of the same symptoms in several subjects with the same pathology is compatible with the non-randomness of associations and interactions between modules.

### Primary progressive apraxia of speech

Apraxia of speech (AOS) is a disorder of the motor planning necessary for the prosodic and phonetic production of speech [60], and should be distinguished from dysarthria, which refers to weakness of the buccophonatory muscles. The earliest descriptions in patients with vascular damage referred to the general concept of oral apraxia (OA), a deficit typically associated with disorders of language production, particularly Broca’s aphasia (see [61], for review and data on vascular patients). However, in two of the patients with left hemisphere damage reported by De Renzi et al. [61], OA was not associated with aphasia, i.e. it represented as an autonomous syndrome. The term AOS was introduced by Darley [62] and is the most commonly used term to define the incoordination of movements necessary for the production of verbal sounds, although there is some terminological inconsistency (see [63] for discussion). The term OA has been retained to refer to an apraxic disorder of the buccophonator apparatus involving extraverbal, i.e. non-speech, movements such as whistling or kissing, which has also been reported by some as a distinct disorder [64]. However, the relationship between aphasia, OA and AOS is not fully defined, mainly because the associations or dissociations observed between these forms could be due to the different extent of the lesion and the different points in the patient’s clinical history at which the disorders are detected. In vascular patients, there is no complete agreement on the location of the lesion causing AOS and OA, especially in the presence of aphasic symptoms. Some studies report the involvement of the prefrontal gyrus and longitudinal fasciculus in AOS and fronto-temporo-parietal and subcortical regions in OA [65], whereas the role of the insula is more controversial [66].

Overall, evidence from neurodegenerative pathologies has provided some support for the observation that AOS can manifest as an autonomous syndrome with respect to the aphasic disorders of primary agrammatic aphasia [67], with neuroanatomical correlates characterised by grey and white matter loss in the premotor and supplementary motor regions bilaterally, but with left hemispheric prevalence. Structural connectivity studies would confirm that AOS is associated with altered diffusivity adjacent to the supplementary motor regions, in a region thus distinct from that associated with primary non-fluent aphasia, in which involvement of the aslant and prefrontal tract is observed [68]. There is also some evidence for the autonomy of OA with respect to AOS, which is associated with bilateral changes in premotor, supplementary motor and prefrontal areas [69].

However, it must be said that although AOS and non-verbal OA can be observed as autonomous syndromes, they are unlikely to remain so throughout the course of the degenerative disease, and the observation of associations in advanced stages of the disease is almost the rule, as is association between the ‘motor’ aspects of production and the actual language impairment of primary non-fluent aphasia [70]. Furthermore, these disorders are predominantly associated with tau pathology. This suggests that AOS and OA are produced by the degeneration of networks that are functionally related and largely interactive to ensure the efficiency of the later stages of verbal production. The actual distinction between movements dedicated to verbal and non-verbal sounds is a critical point still under discussion, since it depends strictly on the assessment modalities followed in the different studies, also in view of the fact that both types of movements largely share a common effector.

Independence from these systems can be attributed to the cortico-bulbar spinal tract, starting from the motor cortex, which ensures the voluntary movements of the muscles controlled by the lower cranial nerves. Foix Chavany syndrome or opercular syndrome was originally described in vascular patients with bilateral damage to the anterior part of the frontal operculum. The existence of a system controlling the voluntary motor aspects of the lower cranial nerves, whose involution leads to a total loss of articulation and phonation (but also of chewing, swallowing and tongue protrusion) with preservation of automatic movements, has been confirmed in neurodegenerative patients with progressive atrophy of the frontal opercular region and the corticobulbospinal tract bilaterally [71–72]. Although this system and the ‘high-level’ control systems of speech production are structurally independent, allowing the emergence of dissociable syndromes, their functional interaction is nevertheless likely, since the primary motor system is also involved in ‘covert’ articulation, which, as a component of the phonological short-term memory system, is engaged in the high-level syntactic processing in production and comprehension [73] (i.e., in language).

Finally, observations reported in neurodegenerative pathologies strongly support the conclusion that linguistic and motor aspects of verbal production should be studied according to an integrated approach [74] taking into account different but highly interactive subsystems/modules.

## Conclusion

Focal neurodegenerative pathologies have provided insights into the role of brain structures that are generally spared from vascular pathology, such as the temporal poles and anterior frontal regions, or subcortical structures. They also suggest that the neural systems that support cognitive functions always have a bihemispheric, albeit asymmetric, distribution. Neurodegenerative pathologies can provide answers to some critical aspects that emerge from studies of patients with focal vascular-type damage carried out according to cognitive neuropsychological models of information processing, but also from functional neuroimaging studies. They legitimise the correspondence between functional and structural modules. As part of a reconsideration of the concept of modularity in the strict sense, they emphasise the associations between symptoms and the interaction between modules/systems rather than the ‘one-way’ of models based on diagrams. They also suggest the usefulness of selecting patients based on the type of pathology, using the case series methodology. The usefulness of observations on focal neurodegenerative diseases as systems pathologies for understanding the mind-brain relationship can be extended to neurodegenerative diseases in general. For example, consider Parkinson’s disease and the importance of the corticostriatal system in cognition and behaviour, or posterior cortical atrophy [75], principally due to Alzheimer’s type pathology, for the identification of the neural bases of spatial attention and visual perception [76].

Observations of patients with neurodegenerative diseases may inspire computational models based on parallel distributed processing, which, unlike those based on information processing, are able to specify the nature of representations and processes, with a potential return for the models.

Functional neuroimaging studies have provided complementary information and have to some extent replaced neuropsychological observation. However, while recognising their role in informing models of the relationship between mind and brain, the study of patients with brain damage, mostly of a neurodegenerative nature, should be given priority over functional neuroimaging studies, especially to legitimise the relationship between specific functions and specific structures (systems).

The Author declares no conflict of interest.
